# Nutrition, Vitamin D, and Calcium in Elderly Patients before and after a Hip Fracture and Their Impact on the Musculoskeletal System: A Narrative Review

**DOI:** 10.3390/nu16111773

**Published:** 2024-06-05

**Authors:** Luisella Cianferotti, Giuseppe Bifolco, Carla Caffarelli, Gherardo Mazziotti, Silvia Migliaccio, Nicola Napoli, Carmelinda Ruggiero, Cristiana Cipriani

**Affiliations:** 1 Bone Metabolic Diseases Unit, Department of Experimental and Clinical Biomedical Sciences, University Hospital of Florence, University of Florence, 50134 Florence, Italy; giuseppe.bifolco@unifi.it; 2 Division of Internal Medicine, Department of Medicine, Surgery and Neuroscience, University of Siena, 53100 Siena, Italy; carla.caffarelli@unisi.it; 3 Department of Biomedical Sciences, Humanitas University, Endocrinology, Diabetology and Andrology Unit, IRCCS Humanitas Research Hospital, 20089 Milan, Italy; gherardo.mazziotti@hunimed.eu; 4 Department of Experimental Medicine, University Sapienza of Rome, 00185 Rome, Italy; silvia.migliaccio@uniroma1.it; 5 Unit of Endocrinology and Diabetes, Department of Medicine, Rome Biomedical Campus University Foundation, 00128 Rome, Italy; n.napoli@unicampus.it; 6 Geriatric and Orthogeriatric Units, Division Gerontology and Geriatrics, Department of Medicine and Surgery, University of Perugia, 06156 Perugia, Italy; carmelinda.ruggiero@unipg.it; 7 Department of Clinical, Internal, Anesthesiological and Cardiovascular Sciences, Sapienza University of Rome, 00185 Rome, Italy; cristiana.cipriani@uniroma1.it

**Keywords:** osteoporosis, fragility fracture, falls, cholecalciferol, calcium carbonate, calcium citrate, calcium intake

## Abstract

Hip fractures are a major health issue considerably impacting patients’ quality of life and well-being. This is particularly evident in elderly subjects, in which the decline in bone and muscle mass coexists and predisposes individuals to fall and fracture. Among interventions to be implemented in hip fractured patients, the assessment and management of nutritional status is pivotal, particularly in subjects older than 65. Nutrition plays a central role in both primary and secondary preventions of fracture. An adequate protein intake improves muscle mass and strength and the intestinal absorption of calcium. Other nutrients with recognized beneficial effects on bone health are calcium, vitamins D, K, and C, potassium, magnesium, folate, and carotenoids. With reference to calcium, results from longitudinal studies showed that the consumption of dairy foods has a protective role against fractures. Moreover, the most recent systematic reviews and meta-analyses and one umbrella review demonstrated that the combination of calcium and vitamin D supplementation significantly reduces hip fracture risk, with presumed higher efficacy in older and institutionalized subjects. Owing to these reasons, the adequate intake of calcium, vitamin D, protein, and other macro and micronutrients has been successfully implemented in the Fracture Liaison Services (FLSs) that represent the most reliable model of management for hip fracture patients. In this narrative review, papers (randomized controlled trials, prospective and intervention studies, and systematic reviews) retrieved by records from three different databases (PubMed, Embase, and Medline) have been analyzed, and the available information on the screening, assessment, and management of nutritional and vitamin D status and calcium intake in patients with hip fractures is presented along with specific prevention and treatment measures.

## 1. Introduction

The prevalence of osteoporosis and related fragility fractures is steadily rising worldwide [1]. Among fragility fractures, hip fractures represent a major health concern, especially in the aging population, namely over the age of 65, and are often the result of falls [2]. Hip fracture is an often-devastating event, likely leading to a hampered quality of life, disability, associated complications, impact on a person’s overall well-being, and increased risk of mortality [3]. Preventative measures and effective treatment are essential for reducing the incidence of these fractures, preventing further fractures, and improving quality of life for those who experience them [4]. Understanding the epidemiology and consequences of hip fractures is essential for healthcare planning, preventive measures, and addressing the specific needs of populations at higher risk.

Public health initiatives and policies aimed at reducing the incidence of hip fractures have to target osteoporosis prevention, fall prevention, and improve the overall well-being of the elderly population. Once a major fragility fracture such a hip fracture has occurred, proper treatment with antiosteoporotic agents becomes mandatory as a secondary prevention strategy for further fractures, the risk of which is imminently high right in the first two years after the first event [5]. Unfortunately, the minority of people who experience a hip fracture are evaluated and begin proper therapy. The treatment gap in osteoporosis refers to the significant discrepancy between the number of people who have osteoporosis or are at high risk of fractures and those who actually receive appropriate diagnosis, treatment, and management for their condition [6]. This gap is a major concern in healthcare for several reasons, contributing to increased rates of fractures, higher healthcare costs, and decreased quality of life for those affected.

Regarding the secondary prevention of fractures, Fracture Liaison Services (FLSs), primarily borne within tertiary clinical centers around the patient with a hip fracture, represent coordinated, multidisciplinary systems designed to identify, diagnose, treat, and manage the care of patients with osteoporotic fractures, with the ultimate goal of preventing subsequent fractures [7]. As a critical component of integrated care for patients suffering from fractures, especially those at risk of osteoporosis or subsequent fractures, the FLS plays a pivotal role in addressing what is often an under-recognized and under-treated condition. Despite the clear evidence of the cost-effectiveness of the FLSs where applied, they are not routinely supported with specific resources by healthcare systems.

**Figure 1:**
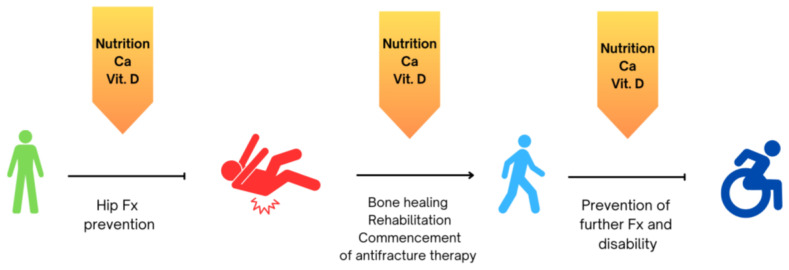
The optimization of nutrition, calcium intake, and vitamin D status is a multimodal approach which can intervene at various stages in the prevention of falls and related hip fragility fractures, recovery from hip fracture, and the prevention of further fractures, along with proper antifracture drugs and related disability.

Nutrition plays a pivotal role in the health and functionality of the musculoskeletal system in elderly individuals, directly impacting their mobility, strength, and risk of falls [8]. The derangement from aging to frailty is often accompanied by an accelerated decline in muscle mass and bone density, leading to a pathological condition known as sarco-osteoporosis. These changes can significantly increase the risk of falls, fractures, and subsequent disabilities, highlighting the importance of nutritional and exercise strategies to mitigate these risks. In particular, nutrition plays a pivotal role in the primary and secondary prevention of osteoporosis and fragility fractures [9] (Figure 1). A good nutritional condition is correlated to better musculoskeletal health in all ages, along with a decrease in the probability of falls and subsequent fractures [9] (Figure 1). Nutritional status is often compromised in elderly people, so that the typical patient with a hip fracture, the risk of which exponentially rises after 65 years of age in both sexes with more than 75% occurring in people older than 75 years, is often nutritionally impaired.

Nutritional status can be evaluated by general nutritional questionnaires, especially formulated for elderly people, as well as vitamin D status and calcium intake assessments. These factors are often neglected in the majority of patients, especially in aged patients after a fragility fracture, so that both patients and care givers are not usually sufficiently educated on the importance of a proper diet and how to manage to achieve it.

The correction of vitamin D status by vitamin D supplementation, even besides baseline vitamin D levels as assessed at the moment of fracture, is mandatory in patients after a major fragility fracture, in order to commence proper treatment early [9]. Indeed, despite the worldwide increase in the consumption of vitamin D supplements in the general population, their use remains low in patients experiencing fragility fractures and, in particular, at the moment of a hip fracture. Mineral intake, and calcium intake in particular, is fundamental for proper bone repair, to optimize the mineralization process and to favor the action of anti-osteoporotic medications. In addition, it has been proven that adverse events, such as hypocalcemia, common for most potent antiresorptives, are less frequent when calcium ingestion is adequate. Calcium intake, which would have always to be optimized in parallel to the improvement in vitamin D status, is usually not systematically evaluated by specific questionnaires, nor have specific interventions been applied systematically in the general management of these patients.

In this review, we will explore the state of the art on the assessment of calcium intake, nutrition, and vitamin D status in patients before and after a hip fracture and analyze whether systematic specific interventions have been undertaken to optimize these parameters in these individuals.

## 2. Methods

For this narrative review, data from three different databases (PubMed, Embase and Medline) were retrieved using the following key words: “hip fracture”, “osteoporosis”, “vitamin D”, “serum 25(OH)D”, “calcium intake”, “nutrition”, “malnutrition”, “protein intake”, “falls”, “disability”, “mortality”, “vitamin D status”, “calcium intake questionnaire”, “elderly”, and “elders”, using “AND”/“OR” operators as appropriate. 

Randomized controlled trials (RCTs), prospective and intervention studies, systematic reviews, and meta-analyses in the English language, mainly focusing on the literature of the last 10 years up to December 2023, were considered. 

Abstracts were reviewed by 3 reviewers (CC, BG, and CL) and only main papers and reviews referring to the prevention of hip fractures and the assessment/treatment of patients with hip fractures and published in English were considered.

## 3. Nutrition and Hip Fracture

In elderly people, a number of physiological factors, such as a loss of appetite, chewing problems, dysphagia, reduced mobility, and psychological problems, and environmental factors, such as mourning, social isolation, and economic problems, can influence eating habits with advancing age [10,11,12].

People’s overall well-being is closely related to nutritional status. Regarding musculoskeletal health, it is crucial to ensure in the elderly an adequate protein intake, as proteins are involved in bone mineral metabolism and are able to improve bone matrix protein collagen [13]. The European Society for Clinical Nutrition and Metabolism (ESPEN) recommended for older adults with acute or chronic illnesses 1.2 to 1.5 g protein/kg body weight/day, increasing the intake in the case of more serious illnesses [14] in order to avoid a progressive loss of lean mass, determined by a nitrogen imbalance [15]. In addition, it appears that an increased protein intake may promote the growth of muscle mass and strength in the lower limbs and increase the intestinal absorption of calcium [16,17].

A poor diet can trigger a range of both physical and mental problems, increasing the risk of conditions such as osteoporosis and falls and recurrent falls [18,19]. These difficulties can contribute to malnutrition due to limited food and fluid intake and due to the difficulty of meeting recommended nutritional requirements. Malnutrition per se is widely recognized for its detrimental effects on various physiological systems, which can lead to increased complications after surgery and increased mortality rates [20]. During the aging process, there is a decrease in the intake of macronutrients and micronutrients, and a decrease in the adherence to a healthy diet, which would allow one to maintain bone mineral mass [21]. Usually, a healthy diet is characterized by a high consumption of whole grains, fruits, vegetables, and legumes that are rich in the micronutrients, such as calcium, vitamin K, potassium, magnesium, vitamin C, folate, and carotenoids, necessary for bone health [22]. Several studies have shown that a high adherence to the Mediterranean diet, ensuring an adequate intake of calcium and vitamin D through food, correlates with a low risk of hip fractures in the elderly [23,24,25,26]. In contrast, other types of different dietary patterns, such as the Western diet, rich in red meat, processed meat, poultry with skin, animal organ meat, cooking oil, soft drinks, hamburgers, hotdogs, ice cream, doughnuts, margarine, and butter [27], and certain nutrients such as saturated fatty acids and trans fatty acids, have been shown to be associated with higher levels of inflammation. Consequently, the inflammatory component of the diet may increase the risk of hip fractures in the elderly population without a gender difference [28,29]. 

As for the role of proteins, scientific evidence demonstrates that a high dietary protein intake is important for fall prevention in old people [30] and it is associated with a low risk of hip fractures [31]. Recently, interesting evidence has been found by Hayashi et al. [32], who show that not only total protein intake but a more frequent consumption of meals with adequate amounts of protein (≥20 g or ≥30 g) may represent a potential strategy to oppose the age-related loss of muscle mass and function, especially in frail and pre-fragile individuals. 

In the UK’s Women’s Cohort Study, including 26,318 women aged 35–69 years, with 822 hip fracture episodes retrieved by hospital records in the >20-year follow-up, an increased intake of protein, calcium, total dairy, and milk was associated with a 45% lower risk of hip fracture for every 25 g/day protein consumed in underweight individuals [33]. In a further analysis of the same cohort, vegetarian women displayed higher risks of hip fracture with respect to regular meat eaters [34]. These findings, although not yet confirmed in males and in other non-European populations, highlight the importance of certain diets and proper nutrition with certain type of foods. 

On admission to a hospital, nursing home, or during outpatient follow-up, as in the case of people experiencing a hip fracture, it is important to assess the patient’s nutritional status. According to the ESPEN guidelines, nutritional status should be diagnosed with standard screening tools, such as the Mini Nutrition Assessment (MNA), Nutrition Risk Screening 2002 (NRS), or the Malnutrition Universal Screening Tool (MUST) [1,35].

Upon admission to the hospital, traditional anthropometric measurements such as weight or body mass index do not allow for the easy identification of the patient. 

Therefore, screening malnutrition in older people with simple tools can be critical for early diagnosis and can direct targeted nutritional interventions to improve postoperative outcomes. According to epidemiological data, malnutrition prevalence in hospitalized elderly patients ranges from 20 to 50% [36]. The prevalence of malnutrition is lower, between 2 and 32%, among the elderly residing with family, and increases with age [10,37]. In addition, one year after hip surgery, patients with malnutrition have a significantly increased risk of mortality [38]. 

Patients admitted to the hospital for a hip fracture showed a low intake of carbohydrates, proteins, fats, certain vitamins, and minerals. The majority of these patients were getting less than the recommended minimum intake, especially for selenium, magnesium, iron, folate, calcium, and zinc [39]. 

The maintenance of good nutritional status in these patients is necessary in order for them to meet their energy requirements. Oral nutritional supplementation (ONS) is generally used during rehabilitation in hip fracture patients with the aim of increasing energy and protein intake, but no convincing effect on mobility, independence, muscle strength, or muscle function has been observed [40].

Several interventions have been proposed to improve nutrition in hip fracture patients, such as not-otherwise-specified enteral nutrition, which seems to have benefits in decreasing postoperative interleukin-6 levels, which could be related to a reduction in time spent in bed and an improved quality of life [41]. Nonetheless, contradictory results are available on the nutritional support via a nasogastric tube, since this procedure has not shown promising results in these patients [42].

In a multicenter randomized controlled trial (RCT), Wyers et al. monitored patients for three months after a hip fracture, with weekly nutritional counselling and intervening with an energy- and protein-rich diet and using ONS [43]. The study showed no effect on lifespan, postoperative complications, or functional parameters, nor on fracture and mortality rates at 1 and 5 years [43]. Recently, Iuliano et al., in another RCT, tested a nutritional intervention with high-calcium dairy foods to increase calcium and protein intake and calcium in institutionalized elderly people, as detailed later in this paper, demonstrating a reduction in falls, total fractures, and hip fractures [44]. 

It seems that interventions that just administer supplements are insufficient and unable to improve clinical outcomes [45]. Indeed, in patients with hip fracture, no positive effects were found even by intervening with just taurine supplementation [46], a semi-essential amino acid with antioxidant action in humans. Bell et al. showed that offering individualized, multidisciplinary, multimodal care involving different professionals such as orthopedists, dietitians, and nursing staff can break down the barriers that cause reduced food intake, and is a key strategy for improving clinical outcomes [47]. Involving a nutritionist and nurses with specific expertise in nutrition has been found to correlate with an increased intake of energy, protein, and supplements [48,49]. These studies demonstrate that a multidisciplinary approach is essential for reducing malnutrition in hospitalized patients. Such a multidisciplinary approach was found to be effective in counteracting the increased incidence of malnutrition after discharge. 

The elderly also experience recurrent falls, which seems to be associated with fatigue, reduced strength, and muscle quality. Therefore, important strategies that are able to work directly on the muscle and preserve it have the potential to improve results. Significant improvements in function and disability reduction in these patients have been achieved by combining structured exercise programs with essential amino acid supplementation [50]. In a cohort study of community-dwelling patients, these conditions appear to be related to nutritional deficiencies and lower hemoglobin levels [51]. Consistent with low hemoglobin levels, there is a reduction in transported oxygen, leading to a reduction in muscle function and occasional falls. 

It seems that one of the strategies to ensure greater patient well-being and improve outcomes is to intervene with a multidisciplinary approach, underlying that fractures are not only an orthopedic issue.

In fact, due to limited access to osteoporosis treatment, several targeted care programs have been designed for patients with this condition and fragility fractures. Programs such as the FLS were implemented to reduce the lack of attention and care for patients with fragility fractures. It seems to result as an effective model; scientific evidence shows that there was increased adherence to medication use, increased exercise, decreased falls, and an increased intake of adequate calcium, vitamin D3, and protein [52,53]. In addition, one-year mortality rates were generally low [54], and the overall re-fracture rate was lower than that reported in previous studies. Currently, the best way to intervene with the dosage of nutritional supplementation, the ideal duration of interventions, and the frequency of nutritional counseling remain unclear. Adherence to the nutritional supplementation regimen and intervention is highly variable, depending on the duration and modality of supplementation [55]. 

## 4. Vitamin D Status and Hip Fractures

Hypovitaminosis D is extremely common in hip fracture patients. The overall prevalence of vitamin D deficiency is reported by studies performed in different countries in the range of 46–92%, depending on the series and thresholds of the serum 25(OH)D used to define deficiency [56,57,58,59,60,61,62,63,64,65,66,67]. Additionally, results from recent systematic reviews and meta-analyses corroborated the hypothesis that low serum 25(OH)D levels represent a risk factor for hip fracture [68,69,70,71,72]. The main results from the meta-analyses published in the last ten years on this topic and on the association between serum 25(OH)D levels and post-fracture outcomes are summarized in Table 1.

Lv et al. reported a pooled relative risk (RR) of hip fracture of 1.58 in association with the lowest vs. the highest serum 25(OH)D levels in a meta-analysis of 13 prospective cohort studies [68]. In particular, subgroup analysis demonstrated that hip fracture risk was increased when low serum 25(OH)D levels were measured in women and subjects older than 65 [68]. Similar results were described by Wang et al. in a meta-analysis of 13 cohort studies in 24,220 patients (2831 with hip fractures) older than 60, in which higher relative risks of hip fracture in association with low 25(OH)D levels were reported, with a RR of 1.25 in female subjects [69]. The analysis of the association between hip fracture risk and the magnitude of serum 25(OH)D decrease showed interesting results, as well. Feng et al. reported a 40% increase in hip fracture risk for any SD decrease in serum 25(OH)D levels in the subgroup meta-analysis of three prospective cohort and case–control studies [72] [2]. Interestingly, Yao et al. described a 20% lower risk of hip fracture for any 10 ng/mL increase in serum 25(OH)D concentration in a meta-analysis of 11 observational studies with 39,141 participants aged 52–76, of whom 2367 had a hip fracture [73]. However, heterogeneity among studies was reported [73]. A more recent systematic review and meta-analysis of 28 studies performed in elderly subjects with a total of 9767 hip fractures clarified that the fracture risk was increased in association with low 25(OH)D serum levels when case–control, cohort, and case–cohort studies from different geographical areas were considered [70].

With reference to post-surgical outcomes, retrospective and prospective studies from several countries collectively showed worse functional recovery in association with lower 25(OH)D concentration [77,78]. A secondary analysis of the Functional Outcomes in Cardiovascular Patients Undergoing Surgical Hip Fracture Repair (FOCUS) Trial including 290 hip fractured subjects 65 and older undergoing surgery between 2004 and 2009 showed reduced mobility in association with vitamin D deficiency before surgery [77]. More specifically, preoperative serum 25(OH)D > 12 ng/mL were associated with 30- and 60-day increased mobility [77]. Subsequent retrospective analyses confirmed the association between pre-surgical vitamin D deficiency and 6- or 12-month worse physical outcomes, re-admission for medical issues, and re-fracture in elderly subjects [78,79,80]. More recently, a meta-analysis of these and other studies in a total of 1972 hip fractured patients aged 78–84 demonstrated that vitamin D deficiency was associated with poorer quality of life and functional ability after fracture, while it did not influence the walking ability and length of stay in the hospital [74].

Retrospective and prospective studies reported inconsistent results as far as the possible role of pre-surgical vitamin D deficiency on mortality, as evaluated from 30 days to 3 years after hip fracture surgery [81]. Llombart et al. performed a meta-analysis of 9 cohort studies in 4409 patients with hip fracture aged 74–87 and concluded that reduced 25(OH)D levels were not associated with overall mortality when adjusting for confounders, such as age or comorbidities. Nevertheless, vitamin D insufficiency [defined as serum 25(OH)D between 20–29.9 ng/mL] and severe vitamin D deficiency [serum 25(OH)D < 10 ng/mL] significantly increased mortality after a year and 60 days from hip fracture surgery, respectively (Table 1) [75]. Wang et al. recently demonstrated significant associations between vitamin D deficiency and mortality but not with functional recovery in hip fractured subjects in a meta-analysis of 18 prospective and retrospective cohort studies (Table 1) [76].

Two recent studies focused on the possible role of vitamin D metabolites and vitamin D binding protein (DBP) on hip fracture risk and post-surgical outcomes. In a post hoc analysis of the Multi-Ethnic Study of Atherosclerosis, Hsu et al. used a composite of first hip and vertebral fracture as the primary outcome and found that total 25(OH)D, PTH, FGF23, and 24,25-dihydroxyvitamin D_3_ to 25-hydroxyvitamin D_3_ ratio were not independently associated with fracture risk in community-dwelling adults [82]. Authors did not report separate analysis for hip fracture in the study and acknowledged that the study was conducted in a population with low fracture risk and high percentage of vitamin D-replete individuals [82]. The post hoc analysis of the FOCUS trial reported significant associations between higher pre-surgical serum DBP levels and better 30- and 60-day mobility and lower 60-day mortality in a total of 260 hip fractured patients with a mean age of 81 [83]. 

In view of data gleaned from observational studies, several intervention studies including RCTs and testing the effect of vitamin D supplementation on hip fracture risk were conducted in different age groups and clinical settings. Owing to these discrepancies, as well as the heterogeneity in vitamin D dose regimens, and, most importantly, in the inclusion of a combination of calcium and vitamin D in the intervention group, results are not consistent. In this context, even results from meta-analyses of these studies failed to find agreement. Table 2 summarizes the results of the meta-analyses published in the last ten years (2014–2023) and including RCTs and non-RCTs studies that evaluated the effect of calcium combined with vitamin D on the risk for hip fractures [73,84,85,86,87,88,89,90,91,92].

The meta-analysis by Yao et al. included 11 RCTs in which supplementation with various dosing regimens of vitamin D (ergo- and cholecalciferol) alone was employed to tests the effects on fracture risk and compared to placebo or no treatment [73]. Authors included RCTs performed in community dwelling as well as in institutionalized subjects [73]. In a total of 34,243 participants aged 66–85 and 740 hip fracture events, no association was detected between vitamin D supplementation and reduction in hip fracture [73]. The same authors performed a meta-analysis of 6 RCTs including 49,282 subjects with a mean age of 66 (730 hip fracture events) treated with daily 400–800 IU of vitamin D and 800–1200 mg of calcium for 6 years on average and demonstrated opposite results [73]. The intervention was indeed associated with a 16% reduction in hip fracture risk compared to placebo or no intervention [73]. Importantly, authors found consistent results between reduction in fracture risk reported in association with serum 25(OH)D levels by observational studies and those observed in the RCTs employing calcium and vitamin D [73]. Manoj et al. specifically meta-analyzed data from seven RCTs performed in subjects older than 65 and supplemented with daily 700–800 IU of cholecalciferol and 500–1200 mg of various calcium compounds [91]. In a total of 12,620 community dwelling and nursing home resident participants, authors reported roughly 25% reduction in hip fracture risk in the calcium and vitamin D combination group compared to placebo or no supplementation [91]. Risk reduction for hip fracture was as high as 30% in association with calcium and vitamin D supplementation when five RCTs performed only in older women were analyzed [91]. The analysis of three studies assessing femoral neck BMD in 483 subjects demonstrated no significant effect of calcium and vitamin D on this specific endpoint [91].

On the other side, the ancillary study of the Vitamin D and Omega-3 Trial (VITAL) failed to find significant effect of vitamin D on fracture risk in midlife and older individuals [93]. The study was conducted in 25,871 healthy adults aged 50 and older of whom only 12.9% had baseline serum 25(OH)D levels < 20 ng/mL and who were not selected for having osteoporosis [93]. Participants were followed up for a median of 5 years, and the daily dose of 2000 IU of cholecalciferol was employed. Compared to placebo, supplementation with cholecalciferol was not associated with any reduction in the incidence of first hip fracture occurring in 56 and 57 subjects in the placebo and vitamin D group, respectively [93]. Similarly to the VITAL, the DO-HEALTH trial reported that the primary prevention through vitamin D supplementation in 1900 healthy, community dwelling subjects older than 70 is not an effective strategy to be pursued to significantly reduce risk for nonvertebral fracture [94,95]. The recent meta-analysis of 18 RCTs including 39,759 subjects older than 50 and with history of fracture by Khatri et al. showed similar results [92]. Besides the observation of no significant effect of vitamin D supplementation alone on fracture risk, the authors reported also no association between calcium and vitamin D intake and reduction of hip fracture (Table 2) [92]. The results were confirmed in the subgroup analysis performed by calcium and vitamin D doses, gender, and baseline serum 25(OH)D levels [92]. 

In the attempt of reducing confusion generated by inconsistent reports published in the last decade on this sensitive topic, an umbrella review of systematic reviews and meta-analyses of RCTs was performed by Chakhtoura et al. [96]. The authors included 12 systematic reviews and meta-analyses assessing the effect of combined calcium and vitamin D on hip fracture risk and 15 on the effect of vitamin D alone. Though the studies by Leboff et al. were not included in the analysis, authors raised important points that collectively confirmed the absence of efficacy of vitamin D supplementation alone on fracture risk; combination of calcium and vitamin D was instead found to be effective for hip fracture risk reduction [96]. Eight systematic reviews and meta-analyses including community dwelling and institutionalized subjects treated with different doses of calcium and vitamin D showed efficacy of the combination on reduction of hip fracture [96]. Two systematic reviews and meta-analyses conducted in community dwelling subjects reported no effect of calcium and vitamin D [96]. The subgroup analysis demonstrated that factors potentially predicting a better response could be age, institutionalization, and baseline serum 25(OH)D < 20 ng/mL, but definitely need further confirmation by specifically designed studies [96].

## 5. Calcium Intake and Hip Fractures

Recovering from a hip fracture requires not just surgical intervention and physical therapy, but also nutritional support to aid the healing process. Calcium plays a crucial role in this recovery phase due to its importance in bone health.

After a hip fracture, the body’s demand for calcium increases to support the repair of bone tissue. Calcium is a key building block for bones; it helps to restore bone density and strength, which is essential for the healing process. Adequate calcium intake can accelerate recovery, reduce the risk of a subsequent fracture, and support overall skeletal health.

The recommended daily calcium intake for older adults is about 1200 mg per day, according to the International Osteoporosis Foundation (IOF), Institute of Medicine and other referral institutions [97,98]. This can be achieved through diet, supplements, or a combination of both. Foods rich in calcium classically include dairy products (such as milk, cheese, and yogurt), green leafy vegetables (like kale and broccoli), fish with edible bones (such as sardines and canned salmon), and fortified foods (like certain cereals and plant-based milk). Chronic administration of drugs such as glucocorticoids or age-related malabsorption decreases intestinal calcium absorption, therefore increasing daily requirement of calcium achievable through diet intervention or supplements.

Although some metanalyses including 33 RCTs involving 51,145 participants, have shown that the increased intake of calcium and/or vitamin D per se is not associated with a lower risk of fragility fractures and hip fractures in particular [86,99], other recent longitudinal studies have shown that consumption of dairy foods, providing proteins and also minerals other than calcium, is protective against fractures. In two large U.S. cohorts, the Nurses’ Health Study (NHS) of women and the Health Professionals Follow-up Study (HPFS) of men, one additional daily serving of total dairy, of which milk contributed about half, was found to be associated with a statistically significant 6% decreased risk of hip fracture among postmenopausal females and men [100]. A more recent analysis of the NHS cohort has further demonstrated that consumption of more than 2 daily servings of total dairy was associated with a decreased fracture risk, including hip fracture, as compared to 1 daily serving (hazard ratio [HR]: 0.74; 95% confidence interval [CI]: 0.61, 0.89) [101]. 

Older individuals are definitely at major risk to have low calcium and protein intake. One recent study examined the impact of dietary sources of calcium and protein on hip fractures and falls in older adults within residential care settings. This cluster RCT focused on increasing dairy food provision as a strategy to enhance calcium and protein intake aiming to reduce fracture and falls [44]. Intervention facilities were assisted by dieticians to increase dairy foods in meals and snacks, with methods such as fortifying milk used in recipes and offering dairy-based desserts. In this study 30 residential care facilities were randomized to provide each individual additional milk, yogurt and cheese to reach a total of 1142 mg calcium/day plus 69 f/day protein calcium, with results on the rate of falls and fractures as compared to care facilities maintaining their usual menu (700 mg/day calcium and 58 g/day protein) [44]. The intervention rapidly reduced the rate of total fractures by 33% and 46% of hip fracture in particular, along with 11% reduction of falls just in the first 5–3 months, demonstrating the effectiveness, promptness and easiness of increasing calcium and protein through dairy foods in preventing falls and fractures in older care residents [44]. An additional paper demonstrated cost-effectiveness of such intervention estimating costs related to fracture management (including ambulance, hospital, rehabilitation and residential care costs) [102].

Total calcium intake of people with fragility fractures is often low, especially in elderly people experiencing a hip fracture, and requires a prompt correction before the commencement of antifracture therapy. Moreover, defective intestinal absorption further decreases the already low calcium intake in the elderly.

Interventions to improve calcium intake and overall nutrition after hip fractures have been explored in various studies, highlighting the potential benefits for recovery and long-term bone health. A systematic review of RCTs evaluated post-surgery interventions for patients with hip fractures, including nutritional supplementation, among various other strategies such as rehabilitation, osteoporosis management, and fall prevention. This review highlighted the diversity of interventions tested and underscored the need for well-rounded postoperative care to improve outcomes in fragile hip fracture patients [103]. The included RCTs considered just calcium and vitamin D supplementation, while interventions to increase nutritional calcium intake by means of dairy products or other calcium sources were not mentioned [103]. Calcium supplements (i.e., calcium carbonate or citrate) along with vitamin D compounds are often easily prescribed, but they are often not tolerated and not taken in the long term.

Few experiences have been published about interventions to raise calcium intake, taking advantage of established FLSs. In a study from Taiwan, calcium intake (by supplements) in patients enrolled in an FLS, including a medication management service to improve medication adherence empowered by follow-up telephone interviews, was significantly improved, as well as vitamin D and protein intake, in the medium–long term, i.e., 1 year after the hip fracture event [53].

Tele-rehabilitation could support multicomponent interventions, such as nutritional intervention and physical exercise, after a hip fracture recovery. In this respect, ongoing RCTs, such as the ActiveFLS RCT, might give us evidence in the future that this approach could improve long-term nutritional status and physical performance, possibly decreasing falls and further fractures after a hip fracture. 

Although it is impossible to dissect and isolate the effect of the improvement of calcium intake on the risk of further fractures, disability, and mortality, the optimization of calcium and vitamin D status is considered mandatory for each antifracture treatment that has to follow the fracture event. 

## 6. Conclusions

The patient with a hip fracture has to be cared for with a multicomponent approach. The importance of nutrition, vitamin D status, and calcium intake is pivotal and should be regarded as a primary resource to be focused on. The target serum 25(OH)D of 30 ng/mL has to be reached and maintained in these patients as recommended by most guidelines [97,104], avoiding high doses, which are likely related to increased falls [105]. Vitamin D pro-hormones, such as cholecalciferol (vitamin D_3_), ergocalciferol (vitamin D_2_), or calcifediol (25(OH)D), can be effectively used to correct vitamin D status before and during antifracture therapy. Besides vitamin D status, which is usually optimized by supplements, calcium intake and overall nutrition should be evaluated and implemented in seniors after a hip fracture, also taking into account their generally suboptimal intestinal absorption. Too few short-term effective interventions have been carried out overall. The medium- and long-term outcomes of persistent changes in nutrition have yet to be assessed.

FLSs, where applied, do not often tackle these issues systematically. Besides nurses and case managers in the FLS, who can be employed to test nutritional status, specific dietary suggestions have to be dispensed by medical personnel or nutrition specialists. By addressing impaired nutrition in elderly individuals, particularly those with hip fractures, healthcare providers can enhance recovery outcomes, reduce complications, and improve the overall quality of life for this vulnerable population. In parallel, the effect of pharmacological intervention to prevent further fractures will be enhanced (Figure 1).

The inclusion of calcium and vitamin D supplementation is considered a potential strategy for improving compromised fracture healing in osteoporotic patients. Clinical evidence suggests that addressing post-traumatic bone loss through such supplementation could be beneficial, especially in the context of the broader management of osteoporosis and fracture healing.

These studies collectively suggest that targeted nutritional interventions, including calcium and protein intake through diet or supplements, along with comprehensive rehabilitation programs, can significantly contribute to improved recovery and mobility after hip fractures. Such strategies, along with targeted physical exercise, are critical for addressing the multifaceted needs of patients recovering from hip fractures, aiming not only at immediate recovery but also at preventing future fractures and enhancing overall bone and skeletal muscle health.

## Figures and Tables

**Table 1:** Summary of the meta-analyses of observational studies assessing the association between serum 25-hydroxy-vitamin D [25(OH)D] levels and hip fracture risk and post-fracture outcomes published from 2014 to 2024.

Author and Year of Publication	Design of Included Studies	Population	Primary Outcome Measure/s	Results
Lv et al., 2017 [68]	Prospective cohort	adults	Association between serum 25(OH)D levels and hip fracture risk	- Significant increased hip fracture risk [RR 1.58 (1.41, 1.77)] in subjects with the lowest vs. the highest serum 25(OH)D levels (15 studies, 51,239 participants); - Subgroup analysis by gender, age, location, type of study, and follow-up confirmed the main results; - The dose–response meta-analysis showed that the increased hip fracture risk is significant when serum 25(OH)D levels are <60 nmol/L.
Feng et al., 2017 [72]	Prospective cohort, case–control	adults	Association between serum 25(OH)D levels and hip fracture risk	- Significant increased hip fracture risk (RR 1.48, 95% CI 1.29–1.68) in subjects with low serum 25(OH)D levels (11 studies); - Subgroup analysis showed 40% increase in hip fracture risk for any SD decrease in serum 25(OH)D levels (RR 1.40, 95% CI 1.20–1.61) (3 studies).
Yao et al., 2019 [73]	Prospective cohort, case–control	adults	Association between serum 25(OH)D levels and hip fracture risk	- Significant 20% decrease in hip fracture risk (RR, 0.80; 95%CI, 0.75–0.86) for any 10 ng/mL increase in serum 25(OH)D levels (11 studies, 39,141 participants); - Subgroup analysis by study design, age, location, follow-up, and baseline 25(OH)D levels showed no significant difference.
Wang et al., 2020 [69]	Prospective cohort	>60 years	Association between serum 25(OH)D levels and hip fracture risk	- Significant increased hip fracture risk [adjusted relative risk (95% CI) 0.89 (0.80, 0.98)] in association with low serum 25(OH)D levels (13 studies, 24,220 participants); - Subgroup analysis by gender, location, and starting time of the study confirmed results for studies performed in Europe, US, and before 2010.
Ghahfarrokhi et al., 2022 [70]	Case–control, cohort, retrospective	elderly	Association between serum 25(OH)D levels and hip fracture risk	- Significant increased hip fracture risk [OR 1.80 (95% CI 1.56–2.07, *p* ≤ 0.001)] in subjects with low vs. those with high 25(OH)D serum levels (28 studies, 61,744 participants); - Subgroup analysis by study design, gender, sample size, follow-up, study period, NOS score, and geographical location confirmed the main results.
Llombart et al., 2023 [74]	Prospective cohort, retrospective cohort	≥50 years	Association between serum 25(OH)D levels and re-habilitation and functional status in subjects with hip fracture	- No significant relationship between the ability to walk and vitamin D deficiency (OR 0.68, 95% CI 0.31–1.53) (3 studies, 586 participants); - No significant relationship between the length of stay in the hospital and vitamin D deficiency (MD 2.27, 95% CI − 2.47 to 7.01) (3 studies, 1395 participants); - Significant relationship between a worse quality of life and functional ability and vitamin D deficiency (SMD −1.50, 95% CI −2.88 to −0.12) (3 studies, 573 participants).
Llombart et al., 2024 [75]	Prospective cohort, retrospective cohort	adults	Association between serum 25(OH)D levels and mortality in subjects with hip fracture	- Significant increased risk of mortality [OR 1.24 (95% CI 1.05–1.46)] in subjects with vitamin D insufficiency (9 studies, 4409 participants); - Analysis adjusted for age, sex, serum albumin levels, and comorbidities showed no significant association between low 25(OH)D levels and mortality; - Subgroup analysis by follow-up time showed significant increased mortality [OR 1.37 (1.06–1.77) and 1.78 (1.01–3.15)] in subjects with vitamin D insufficiency after 1 and 2 years from hip fracture, respectively.
Wang et al., 2024 [76]	Prospective cohort, retrospective cohort	adults	Association between serum 25(OH)D levels and mortality in subjects with hip fracture	- Significant increased risk of mortality [HR 2.29 (95% CI 1.41–3.72)] in subjects with vitamin D deficiency (14 studies); - No significant increased risk of mortality [HR 1.10 (95% CI 0.97–1.24)] in subjects with vitamin D insufficiency (11 studies); - Subgroup analysis by location confirmed the association between vitamin D deficiency and mortality in studies conducted in Europe [HR 2.4 (95% CI 1.28–4.52)], but not in America and Australia; - Subgroup analysis demonstrated no significant association between vitamin D deficiency and recovery of walking ability [HR 1.77 (95% CI 0.95–3.32)].

RR, relative risk; OR, odds ratio; NOS, Newcastle–Ottawa Scale; MDs, mean differences; SMDs, standardized mean differences; HR, hazard ratio.

**Table 2:** Summary of the meta-analyses of randomized controlled trials and clinical studies assessing the effect of the combination of calcium (Ca) and vitamin D (VitD) on hip fracture risk published from 2014 to 2023.

Author and Year of Publication	Design of Included Studies	Population	Setting	Intervention and Comparison	Results
Avenell et al., 2014 [84]	RCTs or quasi-randomized trials	post-menopausal women or men > 65	community or institutional	VitD or VitD-related compounds alone or in combination with Ca vs. placebo, no intervention, or Ca	- Analysis vs. placebo or no treatment (9 trials, 49,853 participants): significant reduction of hip fracture in the Ca/VitD group (RR 0.84, 95% CI 0.74–0.96) - Analysis vs. Ca (7 trials, 7411 participants): no significant reduction of hip fracture in the Ca/VitD group (RR 0.84, 95% CI 0.63–1.13)
Weaver et al., 2016 [85]	RCTs	generally healthy adults	community or institutional	Ca/VitD vs. placebo	- Analysis vs. placebo (6 trials): significant reduction of hip fracture in the Ca/VitD group (SRRE 0.7, 95% CI 0.56–0.87)
Zhao et al., 2017 [86]	RCTs	adults > 50	community	Ca, VitD and Ca/VitD vs. placebo or no treatment	- Analysis vs. placebo or no treatment (7 trials, 8657 participants): no significant reduction of hip fracture in the Ca/VitD group (RR 1.09, 95% CI 0.85–1.39)
Hu et al., 2019 [87]	RCTs	adults > 50	community	Ca, VitD and Ca/VitD vs. placebo	- Analysis vs. placebo: no significant reduction of hip fracture in the Ca/VitD group
Barrionuevo et al., 2019 [88]	RCTs	post-menopausal women with primary osteoporosis or osteopenia at risk for fragility fracture	not specified	various therapies (including Ca/VitD) vs. placebo; head-to-head comparisons	- Analysis vs. placebo: significant reduction of hip fracture in the Ca/VitD group (RR 0.81, 95% CI 0.71–0.93)
Yao et al., 2019 [73]	RCTs	adults	community or institutional	VitD and Ca/VitD vs. placebo or no treatment	- Analysis vs. placebo or no treatment (6 trials, 49,282 participants): significant reduction of hip fracture in the Ca/VitD group (RR 0.61, 95% CI 0.4–0.92)
Eleni et al., 2020 [89]	randomized trials	adults ≥ 50	not specified	Ca/VitD vs. placebo or no treatment	- Analysis vs. placebo or no treatment (8 trials, 68,957 participants): significant reduction of hip fracture in the Ca/VitD group (RR 0.84, 95% CI 0.72–0.97)
Liu et al., 2020 [90]	RCTs	post-menopausal women	not specified	Ca/VitD vs. placebo or no treatment	- Analysis vs. placebo or no treatment (8 trials): significant reduction of hip fracture in the Ca/VitD group (RR 0.86, 95% CI 0.76–0.98)
Manoj et al., 2020 [91]	RCTs	women and men > 65	community or institutional	Ca/VitD vs. placebo or no treatment	- Analysis vs. placebo or no treatment (7 trials, 12,620 participants): significant reduction of hip fracture in the Ca/VitD group (OR 0.75, 95% CI 0.64–0.87)
Khatri et al., 2023 [92]	RCTs	adults ≥ 50 with fracture history	not specified	Ca/VitD vs. placebo or no treatment	- Analysis vs. placebo or no treatment (6 trials, 17,538 participants): no significant reduction of hip fracture in the Ca/VitD group (OR 1.1, 95% CI 0.86–1.4)

RCTs, randomized controlled trials; RR, risk ratio; SRRE, summary relative risk estimate; OR, odds ratio.

## References

[1] Collaborators G.B.D.F. (2021). Global, regional, and national burden of bone fractures in 204 countries and territories, 1990–2019: A systematic analysis from the Global Burden of Disease Study 2019. Lancet Healthy Longev..

[2] Sing C.W., Lin T.C., Bartholomew S., Bell J.S., Bennett C., Beyene K., Bosco-Levy P., Bradbury B.D., Chan A.H.Y., Chandran M. (2023). Global Epidemiology of Hip Fractures: Secular Trends in Incidence Rate, Post-Fracture Treatment, and All-Cause Mortality. J. Bone Miner. Res..

[3] Johnell O., Kanis J.A. (2006). An estimate of the worldwide prevalence and disability associated with osteoporotic fractures. Osteoporos. Int..

[4] Schemitsch E., Adachi J.D., Brown J.P., Tarride J.E., Burke N., Oliveira T., Slatkovska L. (2022). Hip fracture predicts subsequent hip fracture: A retrospective observational study to support a call to early hip fracture prevention efforts in post-fracture patients. Osteoporos. Int..

[5] Legrand M.A., Chapurlat R. (2021). Imminent fracture risk. Jt. Bone Spine.

[6] Compston J. (2020). Reducing the treatment gap in osteoporosis. Lancet Diabetes Endocrinol..

[7] Patel S., Daniels N.F., Lim J.A., Zhou A.K., Thahir A., Krkovic M. (2023). The Importance of Fracture Liaison Services to the Healthcare System: A Review. Curr. Rheumatol. Rev..

[8] Ni Lochlainn M., Cox N.J., Wilson T., Hayhoe R.P.G., Ramsay S.E., Granic A., Isanejad M., Roberts H.C., Wilson D., Welch C. (2021). Nutrition and Frailty: Opportunities for Prevention and Treatment. Nutrients.

[9] Rizzoli R., Biver E., Brennan-Speranza T.C. (2021). Nutritional intake and bone health. Lancet Diabetes Endocrinol..

[10] Kostecka M., Bojanowska M. (2021). An evaluation of the nutritional status of elderly with the use of the MNA questionnaire and determination of factors contributing to malnutrition. A pilot study. Rocz. Panstw. Zakl. Hig..

[11] Sura L., Madhavan A., Carnaby G., Crary M.A. (2012). Dysphagia in the elderly: Management and nutritional considerations. Clin. Interv. Aging.

[12] Krishnamoorthy Y., Vijayageetha M., Kumar S.G., Rajaa S., Rehman T. (2018). Prevalence of malnutrition and its associated factors among elderly population in rural Puducherry using mini-nutritional assessment questionnaire. J. Family Med. Prim. Care.

[13] Bonjour J.P. (2005). Dietary protein: An essential nutrient for bone health. J. Am. Coll. Nutr..

[14] Deutz N.E., Bauer J.M., Barazzoni R., Biolo G., Boirie Y., Bosy-Westphal A., Cederholm T., Cruz-Jentoft A., Krznaric Z., Nair K.S. (2014). Protein intake and exercise for optimal muscle function with aging: Recommendations from the ESPEN Expert Group. Clin. Nutr..

[15] Wolfe R.R. (2012). The role of dietary protein in optimizing muscle mass, function and health outcomes in older individuals. Br. J. Nutr..

[16] Houston D.K., Nicklas B.J., Ding J., Harris T.B., Tylavsky F.A., Newman A.B., Lee J.S., Sahyoun N.R., Visser M., Kritchevsky S.B. (2008). Dietary protein intake is associated with lean mass change in older, community-dwelling adults: The Health, Aging, and Body Composition (Health ABC) Study. Am. J. Clin. Nutr..

[17] Kerstetter J.E., O’Brien K.O., Caseria D.M., Wall D.E., Insogna K.L. (2005). The impact of dietary protein on calcium absorption and kinetic measures of bone turnover in women. J. Clin. Endocrinol. Metab..

[18] Jehu D.A., Davis J.C., Falck R.S., Bennett K.J., Tai D., Souza M.F., Cavalcante B.R., Zhao M., Liu-Ambrose T. (2021). Risk factors for recurrent falls in older adults: A systematic review with meta-analysis. Maturitas.

[19] Verlaan S., Ligthart-Melis G.C., Wijers S.L.J., Cederholm T., Maier A.B., de van der Schueren M.A.E. (2017). High Prevalence of Physical Frailty Among Community-Dwelling Malnourished Older Adults-A Systematic Review and Meta-Analysis. J. Am. Med. Dir. Assoc..

[20] Covinsky K.E., Martin G.E., Beyth R.J., Justice A.C., Sehgal A.R., Landefeld C.S. (1999). The relationship between clinical assessments of nutritional status and adverse outcomes in older hospitalized medical patients. J. Am. Geriatr. Soc..

[21] Movassagh E.Z., Vatanparast H. (2017). Current Evidence on the Association of Dietary Patterns and Bone Health: A Scoping Review. Adv. Nutr..

[22] Yaegashi Y., Onoda T., Tanno K., Kuribayashi T., Sakata K., Orimo H. (2008). Association of hip fracture incidence and intake of calcium, magnesium, vitamin D, and vitamin K. Eur. J. Epidemiol..

[23] Fung T.T., Meyer H.E., Willett W.C., Feskanich D. (2018). Association between Diet Quality Scores and Risk of Hip Fracture in Postmenopausal Women and Men Aged 50 Years and Older. J. Acad. Nutr. Diet..

[24] Warensjo Lemming E., Byberg L., Hoijer J., Larsson S.C., Wolk A., Michaelsson K. (2021). Combinations of dietary calcium intake and mediterranean-style diet on risk of hip fracture: A longitudinal cohort study of 82,000 women and men. Clin. Nutr..

[25] Zeng F.F., Xue W.Q., Cao W.T., Wu B.H., Xie H.L., Fan F., Zhu H.L., Chen Y.M. (2014). Diet-quality scores and risk of hip fractures in elderly urban Chinese in Guangdong, China: A case-control study. Osteoporos. Int..

[26] Benetou V., Orfanos P., Feskanich D., Michaelsson K., Pettersson-Kymmer U., Byberg L., Eriksson S., Grodstein F., Wolk A., Jankovic N. (2018). Mediterranean diet and hip fracture incidence among older adults: The CHANCES project. Osteoporos. Int..

[27] Langsetmo L., Hanley D.A., Prior J.C., Barr S.I., Anastassiades T., Towheed T., Goltzman D., Morin S., Poliquin S., Kreiger N. (2011). Dietary patterns and incident low-trauma fractures in postmenopausal women and men aged >/= 50 y: A population-based cohort study. Am. J. Clin. Nutr..

[28] Shivappa N., Wirth M.D., Hurley T.G., Hebert J.R. (2017). Association between the dietary inflammatory index (DII) and telomere length and C-reactive protein from the National Health and Nutrition Examination Survey-1999-2002. Mol. Nutr. Food Res..

[29] Zhang Z.Q., Cao W.T., Shivappa N., Hebert J.R., Li B.L., He J., Tang X.Y., Liang Y.Y., Chen Y.M. (2017). Association Between Diet Inflammatory Index and Osteoporotic Hip Fracture in Elderly Chinese Population. J. Am. Med. Dir. Assoc..

[30] Zoltick E.S., Sahni S., McLean R.R., Quach L., Casey V.A., Hannan M.T. (2011). Dietary protein intake and subsequent falls in older men and women: The Framingham Study. J. Nutr. Health Aging.

[31] Misra D., Berry S.D., Broe K.E., McLean R.R., Cupples L.A., Tucker K.L., Kiel D.P., Hannan M.T. (2011). Does dietary protein reduce hip fracture risk in elders? The Framingham Osteoporosis Study. Osteoporos. Int..

[32] Hayashi A.P., de Capitani M.D., Dias S.F., de Souza Goncalves L., Fernandes A.L., Jambassi-Filho J.C., de Santana D.A., Lixandrao M., Tavares Dos Santos Pereira R., Riani L. (2020). Number of high-protein containing meals correlates with muscle mass in pre-frail and frail elderly. Eur. J. Clin. Nutr..

[33] Webster J., Greenwood D.C., Cade J.E. (2022). Foods, nutrients and hip fracture risk: A prospective study of middle-aged women. Clin. Nutr..

[34] Webster J., Greenwood D.C., Cade J.E. (2022). Risk of hip fracture in meat-eaters, pescatarians, and vegetarians: Results from the UK Women’s Cohort Study. BMC Med..

[35] Kondrup J., Allison S.P., Elia M., Vellas B., Plauth M. (2003). ESPEN guidelines for nutrition screening 2002. Clin. Nutr..

[36] Inciong J.F.B., Chaudhary A., Hsu H.S., Joshi R., Seo J.M., Trung L.V., Ungpinitpong W., Usman N. (2020). Hospital malnutrition in northeast and southeast Asia: A systematic literature review. Clin. Nutr. ESPEN.

[37] Han T.S., Yeong K., Lisk R., Fluck D., Fry C.H. (2021). Prevalence and consequences of malnutrition and malnourishment in older individuals admitted to hospital with a hip fracture. Eur. J. Clin. Nutr..

[38] van Wissen J., van Stijn M.F., Doodeman H.J., Houdijk A.P. (2016). Mini Nutritional Assessment and Mortality after Hip Fracture Surgery in the Elderly. J. Nutr. Health Aging.

[39] Lumbers M., New S.A., Gibson S., Murphy M.C. (2001). Nutritional status in elderly female hip fracture patients: Comparison with an age-matched home living group attending day centres. Br. J. Nutr..

[40] Kramer I.F., Blokhuis T.J., Verdijk L.B., van Loon L.J.C., Poeze M. (2019). Perioperative nutritional supplementation and skeletal muscle mass in older hip-fracture patients. Nutr. Rev..

[41] Shi H., Lu J.H., Wang S.N., Na Q., Xu L.F., Hong J.A. (2020). Effect of early enteral nutrition in elderly patients with hip fracture during the perioperative period. J. Back. Musculoskelet. Rehabil..

[42] Sullivan D.H., Nelson C.L., Klimberg V.S., Bopp M.M. (2004). Nightly enteral nutrition support of elderly hip fracture patients: A pilot study. J. Am. Coll. Nutr..

[43] Wyers C.E., Reijven P.L.M., Breedveld-Peters J.J.L., Denissen K.F.M., Schotanus M.G.M., van Dongen M., Eussen S., Heyligers I.C., van den Brandt P.A., Willems P.C. (2018). Efficacy of Nutritional Intervention in Elderly After Hip Fracture: A Multicenter Randomized Controlled Trial. J. Gerontol. A Biol. Sci. Med. Sci..

[44] Iuliano S., Poon S., Robbins J., Bui M., Wang X., De Groot L., Van Loan M., Zadeh A.G., Nguyen T., Seeman E. (2021). Effect of dietary sources of calcium and protein on hip fractures and falls in older adults in residential care: Cluster randomised controlled trial. BMJ.

[45] Inoue T., Maeda K., Nagano A., Shimizu A., Ueshima J., Murotani K., Sato K., Tsubaki A. (2020). Undernutrition, Sarcopenia, and Frailty in Fragility Hip Fracture: Advanced Strategies for Improving Clinical Outcomes. Nutrients.

[46] Van Stijn M.F., Bruins A.A., Vermeulen M.A., Witlox J., Teerlink T., Schoorl M.G., De Bandt J.P., Twisk J.W., Van Leeuwen P.A., Houdijk A.P. (2015). Effect of oral taurine on morbidity and mortality in elderly hip fracture patients: A randomized trial. Int. J. Mol. Sci..

[47] Bell J.J., Bauer J.D., Capra S., Pulle R.C. (2014). Multidisciplinary, multi-modal nutritional care in acute hip fracture inpatients—Results of a pragmatic intervention. Clin. Nutr..

[48] Duncan D.G., Beck S.J., Hood K., Johansen A. (2006). Using dietetic assistants to improve the outcome of hip fracture: A randomised controlled trial of nutritional support in an acute trauma ward. Age Ageing.

[49] Hoekstra J.C., Goosen J.H., de Wolf G.S., Verheyen C.C. (2011). Effectiveness of multidisciplinary nutritional care on nutritional intake, nutritional status and quality of life in patients with hip fractures: A controlled prospective cohort study. Clin. Nutr..

[50] Invernizzi M., de Sire A., D’Andrea F., Carrera D., Reno F., Migliaccio S., Iolascon G., Cisari C. (2019). Effects of essential amino acid supplementation and rehabilitation on functioning in hip fracture patients: A pilot randomized controlled trial. Aging Clin. Exp. Res..

[51] Ooi T.C., Singh D.K.A., Shahar S., Rajab N.F., Vanoh D., Sharif R., Tan M.P. (2021). Incidence and multidimensional predictors of occasional and recurrent falls among Malaysian community-dwelling older persons. BMC Geriatr..

[52] Valladales-Restrepo L.F., Castro-Osorio E.E., Ramirez-Osorio J., Echeverry-Martinez L.F., Sanchez-Rios V., Gaviria-Mendoza A., Machado-Duque M.E., Machado-Alba J.E. (2023). Characterization and effectiveness of a Fracture Liaison Services program in Colombia. Arch. Osteoporos..

[53] Chang C.B., Yang R.S., Chang L.Y., Peng J.K., Tsai K.S., Huang W.J., Yang T.H., Chan D.C. (2021). One-year outcomes of an osteoporosis liaison services program initiated within a healthcare system. Osteoporos. Int..

[54] Or O., Fisher Negev T., Hadad V., Shabtai R., Katzir A., Weil Y., Liebergall M. (2021). Fracture Liaison Service for Hip Fractures: Is It A Game Changer?. Isr. Med. Assoc. J..

[55] Dempewolf S., Mouser B., Rupe M., Owen E.C., Reider L., Willey M.C. (2023). What Are the Barriers to Incorporating Nutrition Interventions Into Care of Older Adults With Femoral Fragility Fractures?. Iowa Orthop. J..

[56] Awal W.B.R., Price N., Sadler A., Robinson A., Hymer I., Chen J. (2020). Vitamin D deficiency in proximal femur fracture patients of South-East Queensland. Australas. J. Aging.

[57] Bakhtiyarova S., Lesnyak O., Kyznesova N., Blankenstein M.A., Lips P. (2006). Vitamin D status among patients with hip fracture and elderly control subjects in Yekaterinburg, Russia. Osteop. Int..

[58] Bischoff-Ferrari H., Can U., Staehelin H., Platz A., Henschkowski J., Michel B., Dawson-Hughes B., Theiler R. (2008). Severe vitamin D deficiency in Swiss hip fracture patients. Bone.

[59] Moniz C., Dew T., Dixon T. (2005). Prevalence of vitamin D inadequacy in osteoporotic hip fracture patients in London. Curr. Med. Res. Opin..

[60] Gallacher S.J., McQuillian C., Harkness M., Finlay F., Gallagher A.P., Dixon T. (2005). Prevalence of vitamin D inadequacy in Scottish adults with non-vertebral fragility fractures. Curr. Med. Res. Opin..

[61] Bryson D.J., Neveda J., Ford A.J., Williams S.C. (2013). The incidence of vitamin D deficiency amongst patients with a femoral neck fracture: Are current bone protection guidelines sufficient ?. Acta Orthop. Belg..

[62] Inderjeeth C.A., Barrett T., Al-Lahham Y., Mulford J., Nicklason F., Reberger C. (2002). Seasonal variation, hip fracture and vitamin D levels in Southern Tasmania. N. Z. Med. J..

[63] Dhanwal D.K., Samen S., Gautam V.K., Saha R. (2013). Hip fracture patients in India have vitamin D deficiency and secondary hyperparathyroidism. Osteop. Int..

[64] Phusunti S., Suthutvoravut W., Unnanuntana A., Chotiyarnwong P. (2016). The prevalence of hypovitaminosis D in patient with fragility hip fracture at a single institution in Thailand. J. Med. Assoc. Thai..

[65] Farouk O., Mahran D.G., Said H.G., Alaa M.M., Eisa A.A., Said G.Z., Rashed H., Ez-Eldeen A. (2016). Hypovitaminosis D among patients admitted with hip fracture to a level-1 Trauma Center in the sunny Upper Egypt: Prevalence and associated correlates. Ger. Orthop. Surg. Rehab..

[66] Lakkireddy M., Mudavath S.V., Karra M.L., Arora A.J. (2019). Hypovitaminosis D in patients with osteoporotic hip fractures. J. Clin. Orthop. Trauma..

[67] Ramason R., Selvaganapathi N., Binte Ismail N.H., Wong W.C., Rajamoney G.N., Chong M.S. (2014). Prevalence of vitamin D deficiency in patients with hip fracture seen in an Orthogeriatric Service in sunny Singapore. Ger. Orthop. Surg. Rehab..

[68] Lv Q.B., Gao X., Liu X., Shao Z.X., Xu Q.H., Tang L., Chi Y.L., Wu A.M. (2017). The serum 25-hydroxyvitamin D levels and hip fracture risk: A meta-analysis of prospective cohort studies. Oncotarget.

[69] Wang N., Chen Y., Ji J., Chang J., Yu S., Yu B. (2020). The relationship between serum vitamin D and fracture risk in the elderly: A metaanalysis. J. Orthop. Surg. Res..

[70] Ghahfarrokhi S.H., Mohammadian-Hafshejani A., Sherwin C.M.T., Heidari-Soureshjani S. (2022). Relationship between serum vitamin D and hip fracture in the elderly: A systematic review and meta-analysis. J. Bone Miner. Metab..

[71] Wanby P., Nobin R., Von S.P., Brudin L., Carlsson M. (2016). Serum levels of the bone turnover markers dickkopf-1, sclerostin, osteoprotegerin, osteopontin, osteocalcin and 25-hydroxyvitamin D in Swedish geriatric patients aged 75 years or older with a fresh hip fracture and in healthy controls. J. Endocrinol. Investig..

[72] Feng Y., Cheng G., Wang H., Chen B. (2017). The associations between serum 25-hydroxyvitamin D level and the risk of total fracture and hip fracture. Osteoporos. Int..

[73] Yao P., Bennett D., Mafham M., Lin X., Chen Z., Armitage J., Clarke R. (2019). Vitamin D and calcium for the prevention of fracture. A systematic review and meta-analysis. JAMA Network Open.

[74] Llombart R., Mariscal G., Barrios C., de la Rubia Ortí J.E., Llombart-Ais R. (2023). Does vitamin D deficiency affect functional outcomes in hip fracture patients? A meta-analysis of cohort studies. J. Endocrinol. Investig..

[75] Llombart R., Mariscal G., Barrios C., de la Rubia Ortí J.E., Llombart-Ais R. (2024). Impact of vitamin D deficiency on mortality in patients with hip fracture: A meta-analysis. J. Am. Geriatr. Soc..

[76] Wang K., Xia C., Zhou L., Zheng Y., Wang X., Cheng L. (2024). The Association between Vitamin D Deficiency and the Risk of Mortality after Hip Fractures: A Systematic Review and Meta-Analysis. J. Nutr. Sci. Vitaminol..

[77] Hao L., Carson J.L., Schlussel Y., Noveck H., A Shapses S. (2020). Vitamin D deficiency is associated with reduced mobility after hip fracture surgery: A prospective study. Am. J. Clin. Nutr..

[78] Sim D.S., Tay K., Howe T.S., Koh S.B.J. (2021). Preoperative severe vitamin D deficiency is a significant independent risk factor for poorer functional outcome and quality of life 6 months after surgery for fragility hip fractures. Osteop. Int..

[79] Fu G., Wu R., Zhang R., Chen D., Li H., Zheng Q., Ma Y. (2023). Preoperative vitamin D deficiency is associated with increased one-year mortality in Chinese geriatric hip fracture patients—A propensity score matching study. Clin. Interv. Aging.

[80] Ingstad F., Solberg L.B., Nordsletten L., Thorsby P.M., Hestnes I., Frihagen F. (2021). Vitamin D status and complications, readmissions, and mortality after hip fracture. Osteop. Int..

[81] Nurmi-Lüthje I., Tiihonen R., Paattiniemi E.-L., Sarkkinen H., Naboulsi H., Pigg S., Kaukonen J.-P., Kataja M., Lüthje P. (2022). Relatively low and moderate pre-fracture serum 25-hydroxyvitamin D levels associated with the highest survival in elderly hip fracture patients in Finland: A minimum 3-year follow-up. Osteop. Int..

[82] Hsu S., Criqui M.H., Ginsberg C., Hoofnagle A.N., Ix J.H., McClelland R.L., Michos E.D., Shea S.J., Siscovick D., Zelnick L.R. (2022). Biomarkers of vitamin D metabolism and hip and vertebral fracture risk: The Multi-Ethnic Study of Atherosclerosis. JBMR Plus.

[83] Meng L., Wang X., Carson J.L., Schlussel Y., Shapses S.A. (2024). Vitamin D binding protein and postsurgical outcomes and tissue injury markers after hip fracture: A prospective study. J. Clin. Endocrinol. Metab..

[84] Avenell A., Mecheal J., O’Connell D.L. (2014). Vitamin D and vitamin D analogues for preventing fractures in postmenopausal women and older men. Cochrane Database Syst. Rev..

[85] Weaver C.M., Anna D., Boushey C.J., Dawson-Hughes B., Lappe J.M., LeBoff M.S., Liu S., Looker A.C., Wallace T.C., Wang D.D. (2016). Calcium plus vitamin D supplementation and risk of fractures: An updated meta-analysis from the National Osteoporosis Foundation. Osteop. Int..

[86] Zhao J.G., Zeng X.T., Wang J., Liu L. (2017). Association Between Calcium or Vitamin D Supplementation and Fracture Incidence in Community-Dwelling Older Adults: A Systematic Review and Meta-analysis. JAMA.

[87] Hu Z.C., Tang Q., Sang C.M., Tang L., Li X., Zheng G., Feng Z.H., Xuan J.W., Shen Z.H., Shen L.-Y. (2019). Comparison of fracture risk using different supplemental doses of vitamin D, calcium or their combination: A network meta-analysis of randomised controlled trials. BMJ Open.

[88] Barrionuevo P., Kapoor E., Asi N., Alahdab F., Mohammed K., Benkhadra K., Almasri J., Farah W., Sarigianni M., Muthusamy K. (2019). Efficacy of pharmacological therapies for the prevention of fractures in postmenopausal women: A network meta-Analysis. J. Clin. Endocrinol. Metab..

[89] Elen A., Panagiotis P. (2020). A systematic review and meta-analysis of vitamin D and calcium in preventing osteoporotic fractures. Clin. Rheumatol..

[90] Liu C., Kuang X., Li K., Guo X., Deng Q., Li D. (2020). Effects of combined calcium and vitamin D supplementation on osteoporosis in postmenopausal women: A systematic review and meta-analysis of randomized controlled trials. Food Funct..

[91] Manoj P., Derwin R., George S. (2023). What is the impact of daily oral supplementation of vitamin D3 (cholecalciferol) plus calcium on the incidence of hip fracture in older people? A systematic review and meta-analysis. Int. J. Older People Nurs..

[92] Khatri K., Kaur M., Dhir T., Kankaria A., Arora H. (2023). Role of calcium &/or vitamin D supplementation in preventing osteoporotic fracture in the elderly: A systematic review & meta-analysis. Indian. J. Med. Res..

[93] LeBoff M.S., Chou S.H., Ratliff K.A., Cook N.R., Khurana B., Kim E., Cawthon P.M., Bauer D.C., Black D., Gallagher J.C. (2022). Supplemental vitamin D and incident fractures in midlife and older adults. N. Engl. J. Med..

[94] Bischoff-Ferrari H.A., Vellas B., Rizzoli R., Kressig R.W., da Silva J.A.P., Blauth M., Felson D.T., McCloskey E.V., Watzl B., Hofbauer L.C. (2020). Effect of vitamin D supplementation, omega-3 fatty acid supplementation, or a strength-training exercise program on clinical outcomes in older adults: The DO-HEALTH randomized clinical trial. JAMA.

[95] LeBoff M.S., Bischoff-Ferrari H.A. (2023). The effects of vitamin D supplementation on musculoskeletal health: The VITAL and DO-Health Trials. J. Gerontol. A Biol. Sci. Med. Sci..

[96] Chakhtoura M., Bacha D.S., Gharios C., Ajjour S., Assaad M., Jabbour Y., Kahale F., Bassatne A., Antoun S., Akl E.A. (2022). Vitamin D supplementation and fractures in adults: A systematic umbrella review of meta-analyses of controlled trials. J. Clin. Endocrinol. Metab..

[97] Kanis J.A., Cooper C., Rizzoli R., Reginster J.Y. (2019). Scientific Advisory Board of the European Society for Clinical and Economic Aspects of Osteoporosis and Osteoarthritis (ESCEO) and the Committees of Scientific Advisors and National Societies of the International Osteoporosis Foundation (IOF). Executive summary of European guidance for the diagnosis and management of osteoporosis in postmenopausal women. Aging Clin. Exp. Res..

[98] (1994). Optimal calcium intake. NIH Consens Statement.

[99] Bolland M.J., Leung W., Tai V., Bastin S., Gamble G.D., Grey A., Reid I.R. (2015). Calcium intake and risk of fracture: Systematic review. BMJ.

[100] Feskanich D., Meyer H.E., Fung T.T., Bischoff-Ferrari H.A., Willett W.C. (2018). Milk and other dairy foods and risk of hip fracture in men and women. Osteoporos. Int..

[101] Yuan M., Hu F.B., Li Y., Cabral H.J., Das S.K., Deeney J.T., Zhou X., Paik J.M., Moore L.L. (2023). Types of dairy foods and risk of fragility fracture in the prospective Nurses’ Health Study cohort. Am. J. Clin. Nutr..

[102] Baek Y., Iuliano S., Robbins J., Poon S., Seeman E., Ademi Z. (2023). Reducing hip and non-vertebral fractures in institutionalised older adults by restoring inadequate intakes of protein and calcium is cost-saving. Age Ageing.

[103] Phang J.K., Lim Z.Y., Yee W.Q., Tan C.Y.F., Kwan Y.H., Low L.L. (2023). Post-surgery interventions for hip fracture: A systematic review of randomized controlled trials. BMC Musculoskelet. Disord..

[104] Sanders K.M., Seibel M.J. (2016). Therapy: New findings on vitamin D3 supplementation and falls—When more is perhaps not better. Nat. Rev. Endocrinol..

[105] Bischoff-Ferrari H.A., Dawson-Hughes B., Orav E.J., Staehelin H.B., Meyer O.W., Theiler R., Dick W., Willett W.C., Egli A. (2016). Monthly High-Dose Vitamin D Treatment for the Prevention of Functional Decline: A Randomized Clinical Trial. JAMA Intern. Med..

